# Evaluation of performance of drought prediction in Indonesia based on TRMM and MERRA-2 using machine learning methods

**DOI:** 10.1016/j.mex.2019.05.029

**Published:** 2019-05-28

**Authors:** Heri Kuswanto, Achmad Naufal

**Affiliations:** Department of Statistics, Institut Teknologi Sepuluh Nopember (ITS), Indonesia

**Keywords:** Random forest and CART, Drought, Random forest, CART, Remote-sensing

## Abstract

East Nusa Tenggara Province is one of the most vulnerable regions in Indonesia to drought. Drought prediction is definitely needed as a mitigation action to minimize the risk of drought. However, a sparse dataset has led to difficulties in accurately predicting future droughts in areas without meteorological stations, and hence a dataset with a finer resolution is required. This research investigates the performance of a 3-month Standardized Precipitation Index (SPI) derived from the Tropical Rainfall Measuring Mission (TRMM) and Modern-Era Retrospective analysis for Research and Applications (MERRA-2) to predict drought. CART and Random Forest are applied as the classification methods. Using several predictors, the analysis finds that CART has lower predictability than Random Forest. The average accuracy of the prediction using Random Forest reaches 100% with an average Area Under Curve (AUC) of about 0.8. The analysis also shows that predictions using the MERRA-2 dataset lead to higher accuracy and AUC than those using the TRMM. Therefore, using the MERRA-2 dataset predicted by Random Forest can be an optimal way to predict drought in East Nusa Tenggara. The methods confirmed that average soil surface temperature (day and night), Multivariate ENSO Index (MEI), Arctic Oscillation Index (AOI) and Normalized Difference Vegetation Index (NDVI) are strong predictors of drought. The performance of CART and Random Forest is improved with the Synthetic Minority Over-Sampling Technique (SMOTE).

The techniques described:

• translate drought information and predictors of drought into a base classifier that optimizes the AUC;
• allow drought to be predicted for many grid points efficiently and with high accuracy; and
• are computationally efficient and easy to implement.

## Graphical abstract

**Figure:**
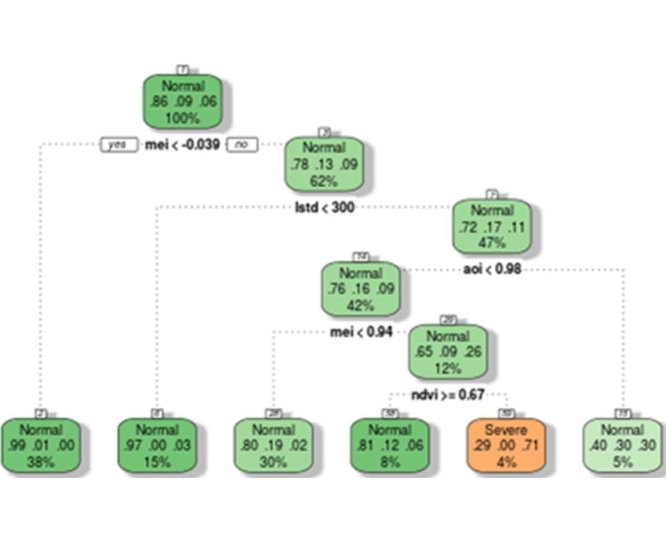

## Specifications Table

**Table:** 

Subject Area:	Environmental Science
More specific subject area:	Drought Prediction
Method name:	Random Forest and CART
Name and references for original method:	Random Forest and CART
	Breiman, L. (1996) Bagging Predictors, Machine Learning, 26, 123-140.
	Breiman, L. (2001) Random Forests. Machine Learning, 45, 5-32.
	Breiman, L. Friedman, J.H., Olshen, R.A., and Stone, C.J. (1984) Classification and Regression Trees, Wadsworth, Monterey, CA.
Resource availability:	MERRA-2 Re-analysis dataset available online
	TRMM satellite data available online
	R (Open source software for data processing)

## Method details

Drought is a natural disaster of below-average precipitation in a certain area caused by disruption to an expected preciptation pattern, and it has a very wide impact. One type of drought that occurs in Indonesia is meteorological drought, which can be defined as an event that takes place naturally and repeatedly because of reduced rainfall from normal conditions [1]. One of the provinces in Indonesia with the most frequent meteorological droughts is East Nusa Tenggara (NTT), and this is listed as the top priority region that is most vulnerable to drought [2]. Mitigation of drought, by methods such as providing reliable predictions of future droughts, is definitely required to minimize the risk and the negative impact of drought.

Meteorological drought monitoring can be done by examining the rainfall data recorded from observation stations [3]; such data are effective and relatively accurate in describing the rainfall in an area. However, meteorological stations are not evenly distributed, resulting in the reduced accuracy of the results of the analysis [4,5], particularly in regions with no stations. To deal with this drawback, a dataset of satellite-based data has been extensively used in many analyses [5]. Remote-sensing data originating from meteorological satellites can provide rainfall data with a better spatial and temporal distribution [6]. According to Rhee and Im [3], remote-sensing data can be used widely and dynamically in drought monitoring. For tropical regions, the remote-sensing data generated from meteorological satellites that have been widely used in various studies are from the Tropical Rainfall Measuring Mission (TRMM) [[7], [8], [9], [10], [11], [12], [13], [14], [15]]. Most studies have found that TRMM satellite precipitation gives a relatively good performance. For the case of Indonesia, the performance of the TRMM has been investigated [16,17]. Hatmoko et al. [18] used TRMM data for drought analysis.

Another dataset that has been extensively used to build drought prediction is Modern-Era Retrospective Analysis for Research and Applications (MERRA-2). This is a re-analysis product that assimilates satellite rainfall estimates from the Special Sensor Microwave Imager (SSMI) and the TRMM Microwave lmager (TMI) [19]. Among the studies that have used MERRA-2 to build drought prediction are that of Kulkani [20], who applied MERRA-2 to the case of India and Chen et al. [21], who compared the performance of MERRA-2 with that of other re-analysis products such as ERA-Interim and the NCEP-2 re-analysis for China, and found that MERRA-2 has a better performance. MERRA-2 has been used in numerous studies in different climatic regions [[22], [23], [24]]. Uncertainties in MERRA-2 datasets have been evaluated against different observations (e.g. [25,26],). The results showed that MERRA-2 provides valuable information consistent with observation, especially in the mid-latitudes, while uncertainties in the high latitudes are often large [27].

This present paper investigates the performance of TRMM and MERRA-2 for predicting drought in East Nusa Tenggara, Indonesia. The prediction of drought will generate a classification of drought, based on the 3-month SPI derived from those two data sources. The 3-month SPI is used because it can describe short- and medium-term humidity conditions, according to the World Meteorological Organization (WMO). In addition, the 3-month SPI has commonly been used by the Indonesian Agency for Meteorology, Climatology and Geophysics (BMKG) for monitoring drought conditions in Indonesia. Following Rhee et al. [3], drought is predicted using several predictors such as the Normalized Difference Vegetation Index (NDVI), the average soil surface temperature day and night, the Multivariate ENSO Index (MEI), and the Arctic Oscillation Index (AOI).

This research applies two different machine learning methods to classify the drought status, Classification and Regression Tree (CART) and Random Forest (RF). Both methods were selected because of their strength in applications to a large sample dataset, as in our case. Moreover, both methods have been proved to be computationally efficient. Various machine learning approaches have been extensively applied in the case of drought prediction (see, for example, [[28], [29], [30], [31], [32]]). The most recent work by Fung et al. [33] provides a comprehensive review of the applications of statistics-based modelling as well as machine learning methods for drought forecasting over the period from 2007 to 2017. Most of the papers agree that machine learning is a powerful tool for drought forecasting. This present paper also proposes the combination of the machine learning methods with sampling method to overcome the problem of imbalance class response as well as to improve the predictive performance.

## Materials and methods

### Data source and variable

The data used in this study are secondary data obtained from several different sources. The remote-sensing data are obtained from https://search.earthdata.nasa.gov. The data cover the spatial region of East Nusa Tenggara, from latitude 8°S to 11°S and longitude 118.75°E to 125.25°E. The SPI is derived from monthly data spanning from 1998 to 2017. The analyses for the two responses (TRMM and MERRA-2) are conducted separately. A short description of the sources of the data follows:

#### Tropical rainfall measuring mission (TRMM)

TRMM or Tropical Rainfall Measuring Mission is a collaborative project between Japan and the United States, especially the space agencies of the two countries, the Japan Aerospace Exploration Agency (JAXA) and the National Aeronautics and Space Administration (NASA).

#### Modern-era retrospective analysis for research and applications (MERRA-2)

The second version of the Modern-Era Retrospective Analysis for Research and Applications is an atmospheric re-analysis that was started by NASA in 1980. MERRA-2 is a re-analysis product, which means that the available data are the result of processing or correcting with certain algorithms.

#### Moderate-resolution imaging spectroradiometer (MODIS)

The surface temperature (day and night) was obtained from MYD11C3 Land Surface Temperature and Emissivity, which is one of the results of the Moderate Resolution Imaging Spectroradiometer (MODIS) sensor on NASA's Aqua satellite. The Normalized Difference Vegetation Index (NDVI) is obtained from the MYD13C2 Vegetation Indices.

#### Multivariate ENSO index (MEI) and arctic oscillation index (AOI)

MEI and AOI are variables that are considered to represent climate conditions globally, especially in predictions of drought. They include information about the anomalies that occur, such as El Nino.

The variables used in this study are in Table 1.

**Table 1:** Research Variables.

Variables	Variable Name	Spatial Resolution	Scale
Y_1_	**SPI-3 TRMM:**	0.25° × 0.25°	Categorical
	≥ (−1.00) = Normal		
	(−1.00) to (−1.49) = Moderate		
	≤ (−1.50) = Severe		
Y_2_	**SPI-3 MERRA-2:**	0.5° × 0.625°	Categorical
	≥ (−1.00) = Normal		
	(−1.00) to (−1.49) = Moderate		
	≤ (−1.50) = Severe		
X_1_	Average surface temperature (Day)	0.05° × 0.05°	Numeric
X_2_	Average surface temperature (Night)	0.05° × 0.05°	Numeric
X_3_	NDVI	0.05° × 0.05°	Numeric
X_4_	MEI	–	Numeric
X_5_	AOI	–	Numeric

### Classification and regression trees (CART)

CART is an algorithm used for classification, and uses a decision tree. The concept behind this method is binary recursive partitioning [34]. There are three stages in classifying using the CART method: forming a classification tree with the formation procedure using recursive node splitting, pruning the trees that are produced to produce a simpler classification tree series, and determining the optimal classification tree.

#### Optimal classification trees

##### Splitting strategy

In the splitting selection, the training data sample is split on the basis of splitting rules and goodness of split criteria, maintaining the heterogeneity of the split samples. The splitting selection depends on the type of tree or on the type of response variable. The results of the splitting process must be more homogeneous than the parent node. The level of heterogeneity of the node can be measured using impurity or r(t). The function of the Gini index is written in the equation as follows:

(1) $$r\left(t\right)=\sum_{c_{0}}^{C_{0}}\sum_{c_{1}}^{C_{1}}p\left(c_{0},t\right)p\left(c_{1},t\right)=1-\sum_{i=0}^{1}{\left(c_{i}\right)}^{2},c_{0}\neq c_{1}$$

where $r\left(t\right)$ is the Gini index (heterogeneity function) at node *t*, $p\left(c_{0},t\right)$ is the proportion of class 0 at node t and $p\left(c_{1},t\right)$ is the proportion of class 1 at node t. Furthermore, the criteria for goodness of split are determined with a splitting evaluation carried out for split *s* at node *t*. The formula for calculating the value of goodness of split is the following:

(2) $$\phi \left(s,t\right)={\text{Δ}}_{1}\left(s,t\right)=r\left(t\right)-p_{L}r\left(t_{L}\right)-p_{R}r\left(t_{R}\right)$$

where $\phi \left(s,t\right)$ is the value of the goodness of split, $r\left(t\right)$ is the heterogeneity function at node *t*, $p_{L}$ and $p_{R}$ are the proportion of the right node observations on the left and right sides, respectively, and $r\left(t_{L}\right)$ and $r\left(t_{R}\right)$ are the heterogeneity functions at the right and left nodes. The split that produces the highest value of goodness of split is the best split because it can reduce heterogeneity further. Each variable will produce a score to show how much the variable contributes to the tree formation process.

##### Terminal nodes

A node t is a terminal node when there is no significant decrease in heterogeneity, or there is only one observation at each child node, or there is a minimum limit of observations m for each child node produced.

##### Class label

Marking class labels on the terminal nodes based on the rules of the highest number is shown in the following equation:

(3) $$p\left(c_{i},t\right)=\text{m}\text{a}\text{x}\;p\left(c_{i},t\right)$$

The class label for the terminal node t is *c_i_* which gives the expected value of classifying errors at the smallest node *t*, which is equal to $\text{r}\left(\text{t}\right)=1-\text{m}\text{a}\text{x}\;p\left(c_{i},t\right)$.

#### Classification tree pruning

Pruning the classification tree, commonly called pruning, needs to be done because the more splitting that is done, the smaller the level of prediction errors, or, in other words, the prediction value exceeds the actual value (overfitting). Tree pruning is done by determining the minimum cost of complexity. The cost complexity value can be calculated by the following equations:

(4) $$R_{a}\left(T\right)=R\left(T\right)+a\left|\tilde{T}\right|$$

where $R_{a}\left(T\right)$ denotes a measure of the complexity of a tree T on complexity a, $R\left(T\right)$ is the tree resubstitution estimate or misclassification rate of *T* trees, a is the cost complexity parameter for adding a terminal node to the *T* tree, and $\left|\tilde{T}\right|$ is the number of terminal nodes in the *T* tree.

#### Optimal classification tree determination

The replacement estimator is often used if there are a large of observation in the test sample. This procedure is applied by dividing the sample L into two parts, $L_{1}$ (*training*) and $L_{2}$ (*testing*). The observations in $L_{1}$ are used to form T trees, while the observations in *L*_2_ are used to estimate *R(T).* N_1_ is the number of observations in $L_{1}$ and ${\text{N}}_{2}$ the number of observations in $L_{2}.\;$ Furthermore, $\text{X}\;\left(.\right)$ is 0 if the statement in parentheses is wrong and is 1 if the statement in parentheses is correct. The test sample estimator can be shown in the following equation:

(5) $$R^{ts}\left(T_{t}\right)=\frac{1}{N_{2}}\sum_{\left(x_{n},j_{n}\right)\in L_{2}}^{N}X(d(x_{n})\neq j_{n})$$

where $R^{ts}\left(T_{t}\right)$ is the total proportion of errors in the test sample estimate, and $N_{2}$ is the number of observations in the L_2_ training data. In this case we want to estimate the proportion of errors generated from the classification tree formation process, so that the optimal classification tree chosen is the $T_{t}$ tree which has the minimum test sample estimation value or $R^{ts}\left(T_{t}\right)={\text{min}}_{t}R^{ts}\left(T_{t}\right)$.

### Random forest

The Random Forest method is a development of the CART method that applies the bootstrap aggregating (bagging) and random feature selection methods [35,36]. In this method many trees are made so that a forest is formed, and the following analysis is performed on the trees:

1 Perform a random sample size *n* with replacement in the data. This is the bootstrap stage.
2 Using a bootstrap sample, the tree is built until it reaches the maximum size (without pruning). Tree construction is carried out by applying random feature selection in each split selection process, that is, *m*, the predictor variable, is chosen randomly, where *m* << *p*, then the best split is selected based on the predictor variable *m*.
3 Repeat steps 1 and 2 *B* times, so that a forest consisting of *B* trees is formed.

### Evaluation of classification results

Area Under Curve is the area under the curve of the ROC or receiver operating curve. In general, AUC is used for classification problems in binary data; by binarizing, the AUC can be obtained by calculating the average for all combinations of AUC one-against-one, and this has the same function as AUC in general [37]. The classification evaluation is performed by AUC average based on the cross tabulations in Table 2.

**Table 2:** Cross Tabulation of Classification Results.

Actual Class	Predicted Class			Total
	0	1	2	
0	*m_00_*	*m_01_*	*m_02_*	*M_0._*
1	*m_10_*	*m_11_*	*m_12_*	*M_1._*
2	*m_20_*	*m_21_*	*m_22_*	*M_2._*

Total	*M_.0_*	*M_.1_*	*M_.2_*	*M*

where:

*m_ij_* = the number of observations of class *i* rightly predicted as belonging to class *j* (*i = j*).

*m_ij_* = the number of observations from class *i* incorrectly predicted as belonging to class *j*. (*i* ≠ *j*).

*M_i._* = number of observations of class *i*.

*M_.j_* = number of observations of class *j*.

*M* = total number of observations or predictions.

The accuracy can be calculated by dividing the number of observations classified correctly by the total number of observations. The formula for calculating the AUC in binary classification and AUC in multiclass classification is as follows:

$$\hat{A}\left(c_{i}|c_{j}\right)=\frac{1}{mn}\sum_{i=1}^{m}\sum_{j=1}^{n}\text{Ψ}(\rho_{i}>\rho_{j})$$

Ψρi>ρj=1, ρi>ρj12, ρi=ρj0,ρi<ρj 

$$AUC\left(c_{i},c_{j}\right)=\frac{\hat{A}\left(c_{i}|c_{j}\right)+\hat{A}\left(c_{j}|c_{i}\right)\;}{2}$$

$${AUC}_{total}=\frac{2}{C(C-1)}\sum_{i<j}AUC(c_{i},c_{j})$$

where

$\rho_{i}$ = Opportunities for an observation with *k* positive class to be classified into a positive class.

$\rho_{j}$ = Opportunities for an observation with *l* negative class to be classified into a positive class.

*m* = The number of positive class observations.

*n* = The number of negative class observations.

C = Number of classes in the multiclass classification.

## Validation method

**Fig. 1:**
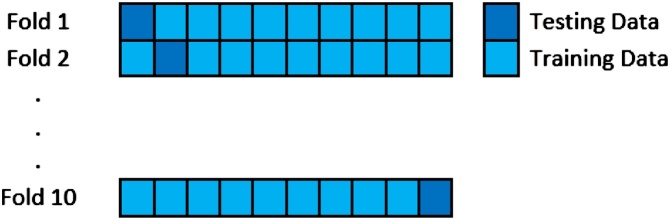
Illustration of the 10-fold cross validation procedure.

The validation method used in this analysis is k-fold cross validation. In k-fold cross validation, the sample data are divided randomly into a number of parts, with each part having equal proportions, and this is repeated many times. The k value that is often used is 10, because it is the value that gives the best estimate of error [38]. An illustration of data sharing using this validation method is found in Fig. 1.

## Results and discussions

**Fig. 2:**
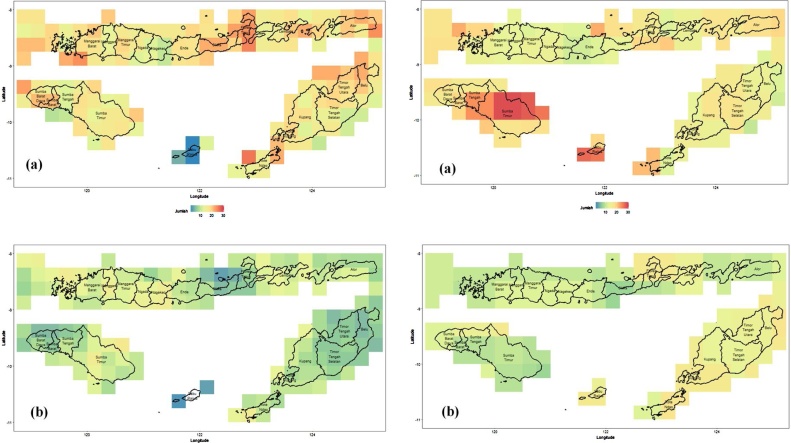
Drought characteristics in East Nusa Tenggara Province based on TRMM (left) and MERRA-2 (right) for (a) moderate and (b) severe levels.

Prior to the analysis, the data were pre-processed to obtain the same grid resolution for all variables i.e. 0.25° x 0.25°. Therefore, the resolution of the pixel in the maps is about 27.5 km x 27.5 km. Fig. 2 shows the number of months in which droughts in NTT were detected from the TRMM (left) and MERRA-2 (right) data. The upper panel of the figure shows the occurrence of moderate droughts, while the lower panel shows the number of severe droughts. Using the data from July 2002 until August 2018, based on the 3-month SPI data derived from TRMM and MERRA-2, it can be revealed that a drought happened almost every year in NTT, at either a moderate or a severe level. From the 3-month SPI, we see that the MERRA-2 data overestimate the TRMM data in all cases. This is shown by the number of drought occurrences, where the TRMM data show a lower number of occurrences of moderate and severe droughts than MERRA-2. On average over the whole area, NTT experienced about 15 to 20 months of moderate drought and about 10 months of severe drought, within these 16 years. Furthermore, MERRA-2 shows that the south-eastern part of NTT experienced a longer drought than the other regions (more than 10 months of drought in total).

### Drought level classification based on 3-month SPI using the CART method

**Fig. 3:**
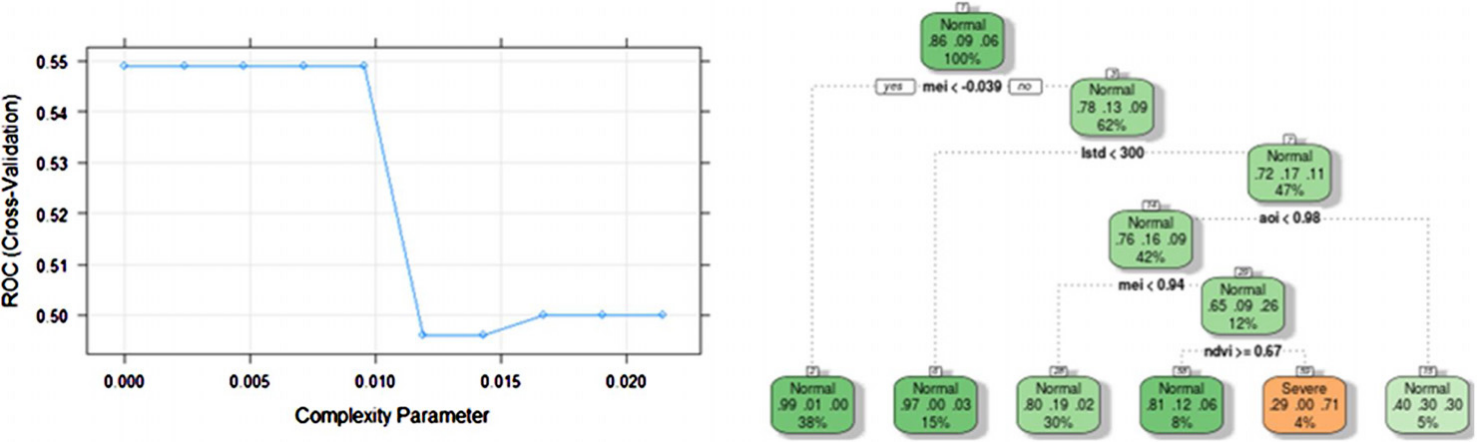
Complexity parameter (left) and tree (right) at the selected grid.

The analysis is done on a grid basis, meaning that the analysis for one grid (we occasionally refer to an area) is independent of the analysis for another. An example is given for the CART analysis at longitude 120.125°E and latitude 8.625°S. Fig. 3 shows the determination of the optimal complexity parameter as a step in CART for pruning the classification tree. We see from the figure that the optimum complexity parameter is 0.0095. The classification tree in Fig. 3 can be used to predict the drought level in the specified grid. Suppose that at a certain condition where X4 = 1, X1 = 400, X6 = 0.5, and X3 = 0.68, the drought level is classified into class 2 (moderate).

Using the optimum complexity parameter, we obtain the AUC and accuracy for both the training and the testing dataset as shown in Table 3.

**Table 3:** Accuracy and AUC of CART for drought prediction using TRMM at (8.625°S; 120.125°E).

Fold	Training Accuracy (%)	Training AUC	Testing Accuracy (%)	Testing AUC
Fold01	85.71%	0.5000	84.21%	0.5000
Fold02	85.23%	0.5000	88.89%	0.5000
Fold03	85.63%	0.5000	85.00%	0.5000
Fold04	86.78%	0.7806	80.00%	0.6458
Fold05	86.78%	0.8690	80.00%	0.8824
Fold06	85.23%	0.5000	88.89%	0.5000
Fold07	86.78%	0.7933	70.00%	0.5980
Fold08	86.29%	0.8401	73.68%	0.4363
Fold09	85.63%	0.5000	85.00%	0.5000
Fold10	86.78%	0.8661	80.00%	0.4265

Average	86.08%	0.6649	81.57%	0.5489

**Fig. 4:**
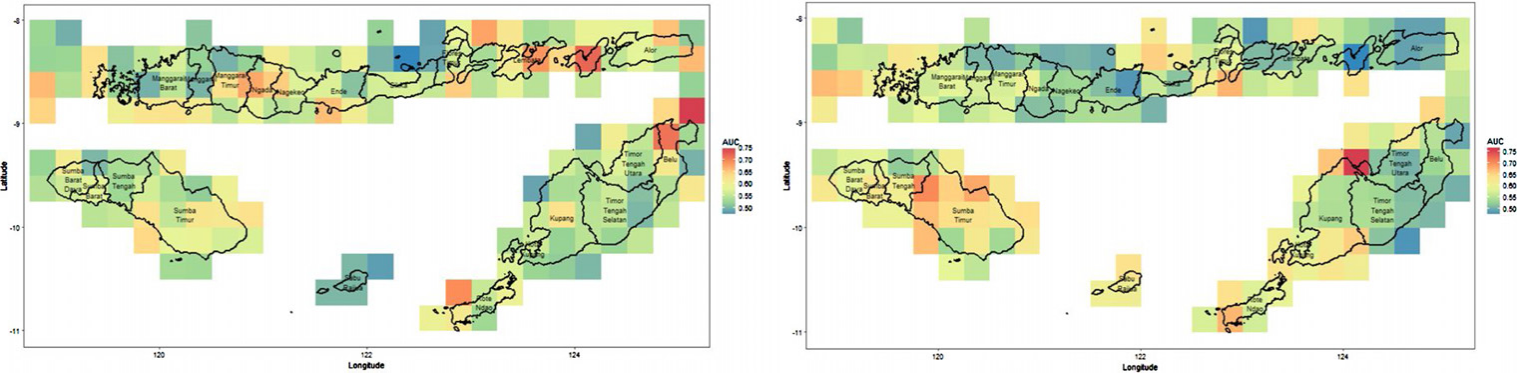
Plot of AUC for TRMM (left) and MERRA-2 (right) analysed using CART.

The table reveals that the CART method is able to predict drought in this area with an accuracy of above 80%. However, the AUCs are very low for both the training and the testing dataset. The high accuracy comes from the unbalanced class response, while the AUC considers this balancing issue in the formula. The process above is repeated for all grids, and results in average AUC values as plotted in Fig. 4. Note that we used 10 cross validations (folds) for the CART analysis. The left side is the AUC for drought prediction using the TRMM dataset, while the right side is the AUC for drought prediction using the MERRA-2 dataset.

From Fig. 4, it is known that CART can classify the drought level with AUC of 0.5 to 0.75. Both the TRMM and the MERRA-2 datasets produce similar AUCs, although there are some inconsistencies in one particular region.

### Drought level classification based on 3-month SPI using random forest method

**Fig. 5:**
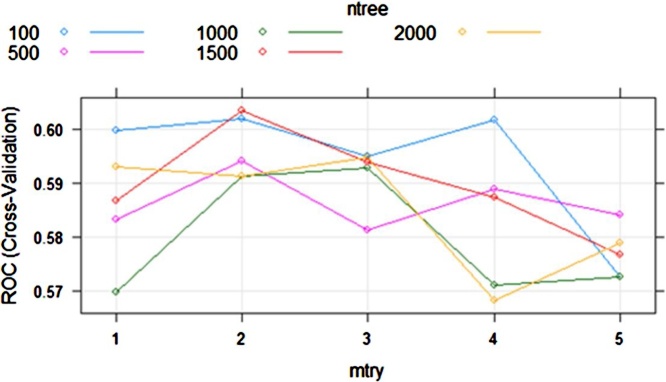
Settings of parameters for Random Forest.

The analysis using Random Forest is carried out as follows. We set the parameters mtree = 1, 2, 3, 4, 5 and ntry = 100, 500, 1000, 1500, 2000 and evaluate the AUC mean value obtained from a 10 cross validations procedure, similar to the analysis with CART. A sample of the analysis step is given for the same grid as with the CART method. Fig. 5 below depicts the tuning parameter of the Random Forest.

We see from the figure that the combination of mtree of 2 with ntry of 1500 is the optimal setting to predict drought in this area (grid), resulting in an AUC of 0.6033. Table 4 shows the accuracy and AUC for each fold. We see that the average AUC in this grid reaches 0.6, which is significantly higher than the one obtained with CART.

**Table 4:** Accuracy and AUC of Random Forest for drought prediction using MERRA-2 at (8.625°S; 120.125°E).

Fold	Training Accuracy (%)	Training AUC	Testing Accuracy (%)	Testing AUC
Fold01	86.29%	0.6036	84.21%	0.4896
Fold02	86.36%	0.5830	88.89%	0.6146
Fold03	84.48%	0.6149	85.00%	0.7696
Fold04	85.06%	0.6120	80.00%	0.5469
Fold05	85.06%	0.5908	90.00%	0.8971
Fold06	84.66%	0.5923	88.89%	0.5885
Fold07	86.21%	0.6139	85.00%	0.3186
Fold08	85.14%	0.6412	84.21%	0.3627
Fold09	86.21%	0.5889	85.00%	0.8284
Fold10	86.21%	0.5898	85.00%	0.6176

Average	85.57%	0.6030	85.62%	0.6034

**Fig. 6:**
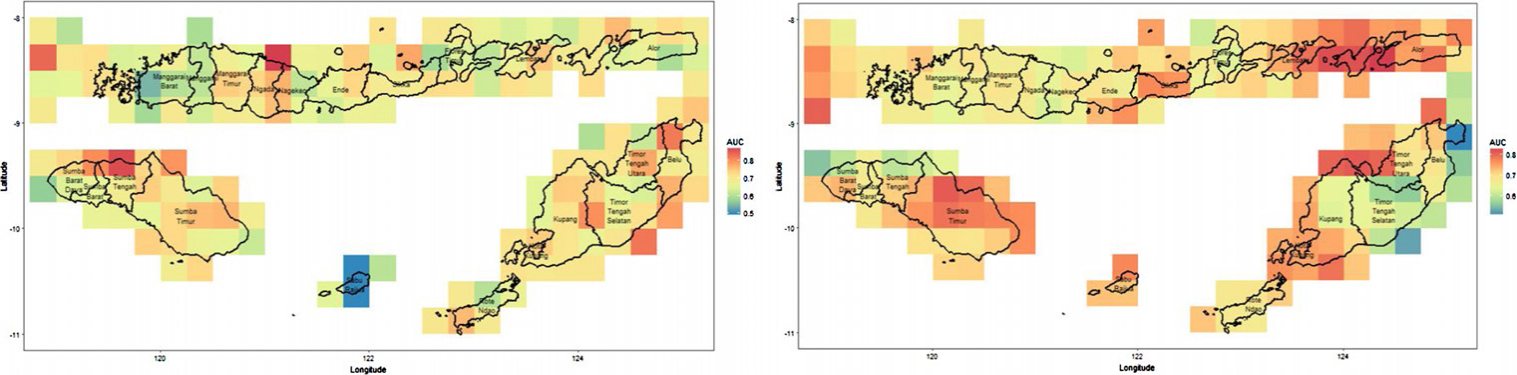
Plot of AUC for TRMM (left) and MERRA-2 (right) analysed using Random Forest.

The step above is then repeated for all grids. Note that the optimum parameters above are valid only for that area, and the parameters can be different for other areas. The results of the average AUC for all grids are depicted in Fig. 6.

From the figure, we see that drought prediction using MERRA-2 yields significantly better AUC figures than prediction using TRMM, as shown by the proportion of areas with an AUC higher than 0.8. However, if we compare the results of the analyses using CART and Random Forest, we can clearly see that the Random Forest improves the accuracy and the AUC significantly. Overall, the average accuracy of drought prediction in NTT using Random Forest reaches 80%.

### Improving the prediction performance using Synthetic Minority Over-Sampling Technique (SMOTE)

The results presented above indicated that both CART and Random Forest have modest performance in particular of the AUC values. The AUC closes to 0.5 indicates that the method tends to predict the majority class e.g. similar to a random guess. Therefore, the prediction performance needs to be improved. One of the very obvious reasons of the low AUC is the imbalance response class. Note that there were only about 15% “very dry” condition found within the examined periods and it creates imbalance response classes, which is an essential issue in classification problem. To overcome this problem, this section proposes to improve the prediction performance by combining the machine learning methods with Synthetic Minority Oversampling Technique (SMOTE). We denoted hereafter the methods as SMOTE-CART and SMOTE-Random Forest, for the combination of oversampling with CART and Random Forest respectively.

**Fig. 7:**
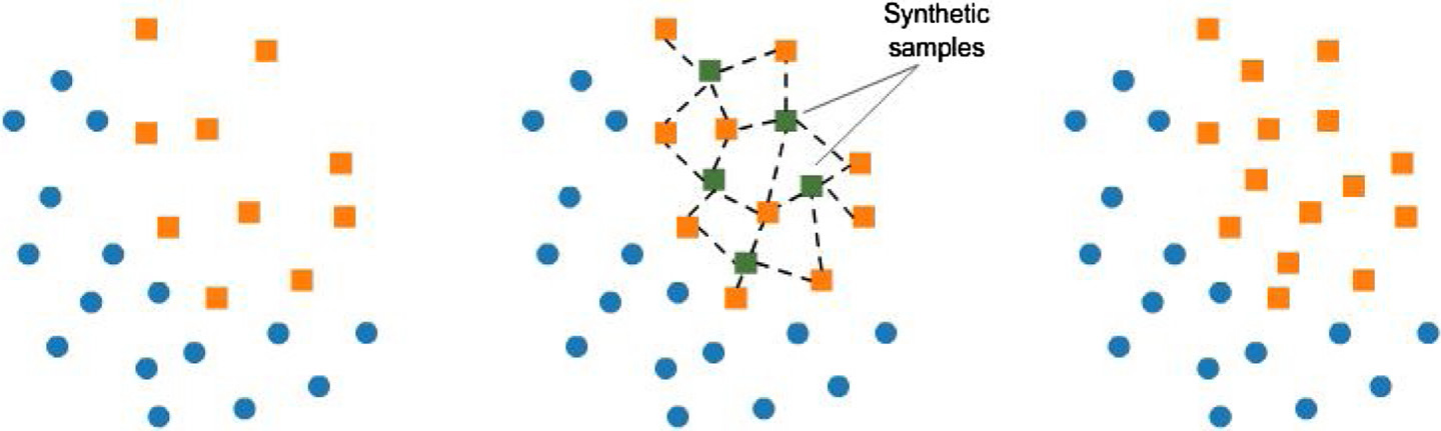
Illustration of the SMOTE Procedure.

The SMOTE is one of the methods for controlling imbalance data proposed by Chawla et al. [38]. The basic idea of SMOTE is to increase the number of samples in the minor class to be equivalent to the major class by generating synthetic data based on the nearest k-nearest neighbor where the closest neighbor is chosen based on the euclidean distance between the two data. The illustration of SMOTE procedure is given in Fig. 7.

**Fig. 8:**
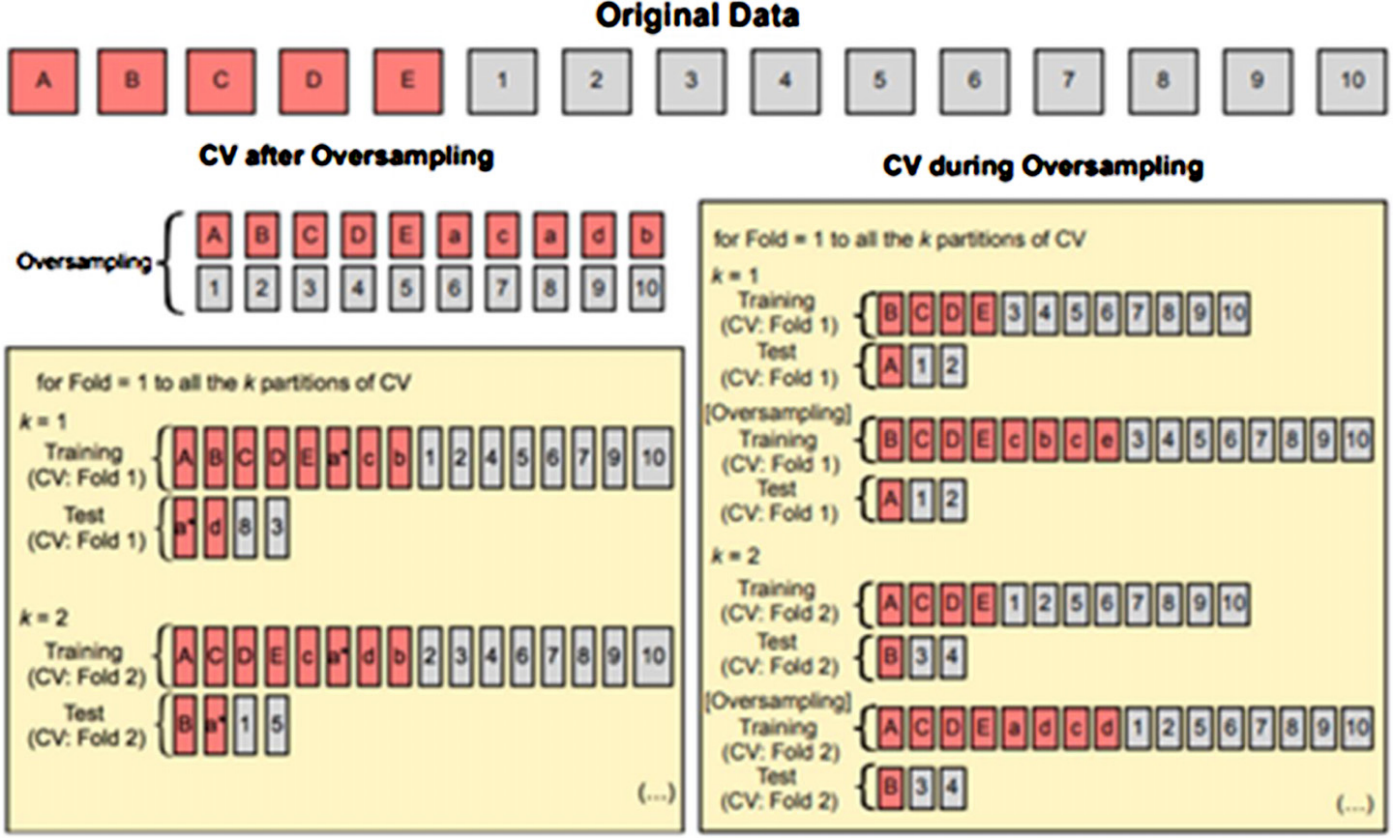
Illustration of SMOTE procedure in *k*-fold cross validation.

Given a dataset with *r* variable i.e. $x^{T}=\left[{\text{x}}_{1},{\text{x}}_{2},\ldots ,{\text{x}}_{r}\right]$ and $z^{T}=\left[{\text{z}}_{1},{\text{z}}_{2},\ldots ,{\text{z}}_{r}\right]$ the eucledian distance *d*(x,z) can be calculated by $d\left(\text{x},\text{z}\right)=\sqrt{{\left({\text{x}}_{1}-{\text{z}}_{1}\right)}^{2}+{\left({\text{x}}_{2}-{\text{z}}_{2}\right)}^{2}+\ldots +{\left({\text{x}}_{r}-{\text{z}}_{r}\right)}^{2}}$. The synthetic data generation is done by using the following equation:

(5) $$x_{syn}=x_{i}+(x_{knn}-x_{i})\gamma$$

where $x_{syn}$ is the synthetic data, $x_{i}$ is the *i-th* data from the minor class, $x_{knn}$ is data with the closest distance from the data to be replicated and γ is random numbers between 0 and 1. The SMOTE will be run under *k*-fold cross validation for each training data. It is done to avoid overoptimistic results due to the pattern replication on training and testing data if the sampling is applied to the entire data [39]. The illustration of SMOTE procedure in *k*-fold cross validation is given in Fig. 8.

**Fig. 9:**
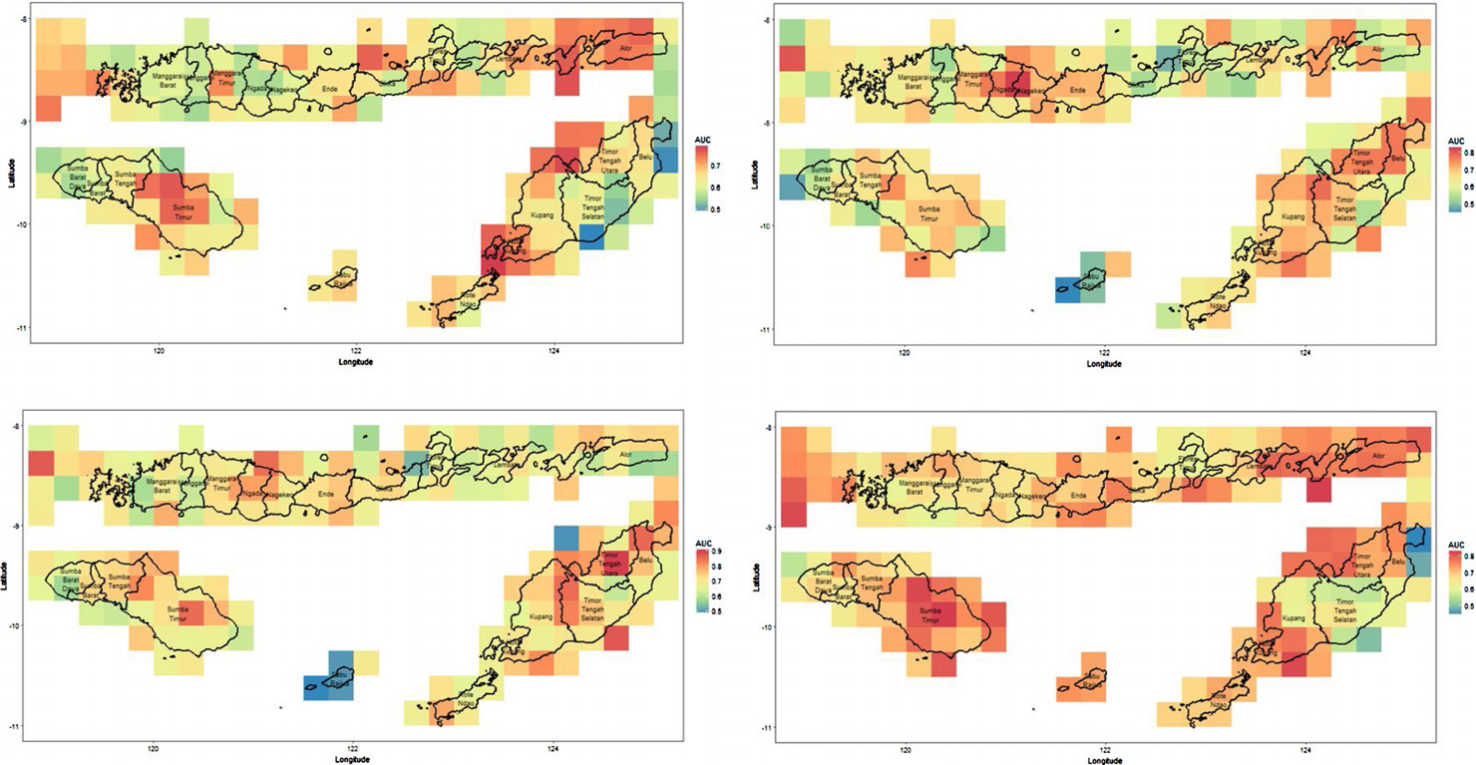
Performance of SMOTE-CART (upper panel) and SMOTE-Random Forest (lower panel) with TRMM (left) and MERRA-2 (right).

This part mainly focuses on improving the AUC which represents the classification performance overall. The results of predicting drought in NTT based on TRMM and MERRA-2 using SMOTE-CART and SMOTE-Random Forest can be seen Fig. 9.

**Fig. 10:**
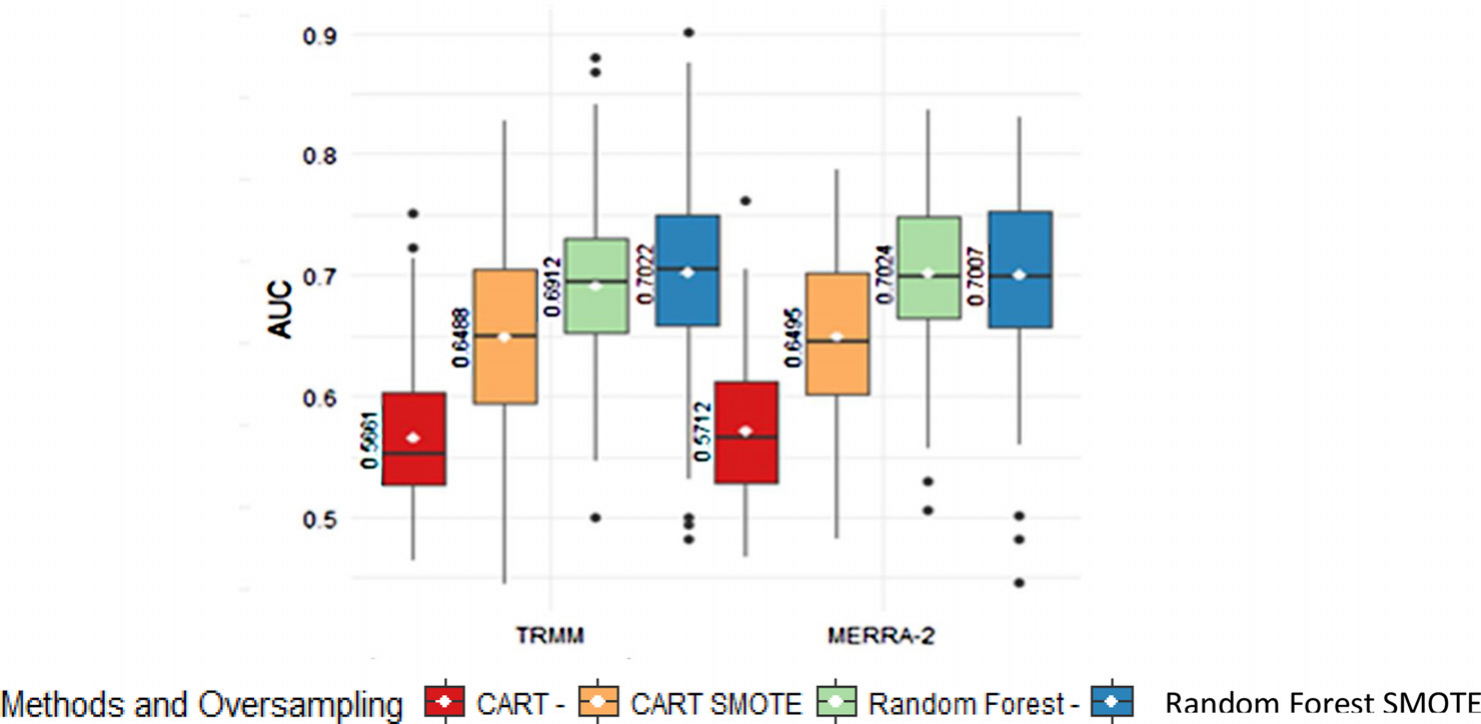
Summary statistic of the AUC values of CART and Random Forest with and without SMOTE.

If we compare the CART performance in Fig. 4 with SMOTE-CART performance in the upper panel of Fig. 9, we observe a significant improvement on the AUC values overall. Meanwhile, the Random Forest performance in Fig. 6 with SMOTE-Random Forest in the lower panel of Fig. 9 are relatively similar with only slight improvement. Increasing the AUC values means that the drought predictability at the corresponding region is significantly improved. In some regions, the classification accuracy exceeds 90%. To summarize, the comparison can be seen in Fig. 10.

The boxplots in Fig. 10 present the AUC values over the entire regions in NTT. We see that SMOTE improves CART performance significantly, both for TRMM and MERRA-2 data. In all cases, Random Forest is robust against imbalance response issue and it still outperforms CART either with SMOTE or without SMOTE. Interestingly, the SMOTE slightly improves the Random Forest performance if we focus only on the mean of the AUC. Furthermore, if we look at the AUC distribution entirely, some regions show higher AUC after sampling.

## Conclusion

Drought prediction analysis in East Nusa Tenggara was performed using two different data sources (TRMM and MERRA-2) and two different machine learning methods (CART and Random Forest). The analysis showed that there is no significant difference in performance between TRMM and MERRA-2, when the drought prediction is carried out using CART. The average AUC reached a maximum of 0.75. Meanwhile, the analysis using Random Forest significantly improved the AUC of the prediction, with the AUC reaching 0.8. Unlike with CART, the drought prediction accuracy using TRMM was significantly different from that with MERRA-2 when the analysis used Random Forest. In this case, MERRA-2 outperformed TRMM. Although many studies have shown that no single machine learning method will always perform better than the others, this study supports the fact that Random Forest is a very powerful method. Moreover, the analysed datasets clearly have imbalance responses, which is an important issue in machine learning applications. To deal with this, the drought prediction accuracy can be improved by applying a certain method to overcome the imbalance in the response class i.e. oversampling (SMOTE), prior to the classification. The SMOTE improves the CART performance significantly, while the Random Forest performance is slightly improved after SMOTE. To conclude, we would suggest that the MERRA-2 dataset, predicted using Random Forest, is used to obtain more accurate drought prediction in East Nusa Tenggara, Indonesia.
